# Effects of Response Option Order on Likert-Type Psychometric Properties and Reactions

**DOI:** 10.1177/00131644211069406

**Published:** 2022-01-13

**Authors:** Chet Robie, Adam W. Meade, Stephen D. Risavy, Sabah Rasheed

**Affiliations:** 1 Wilfrid Laurier University, Waterloo, Ontario, Canada; 2 North Carolina State University, Raleigh, USA

**Keywords:** response option order, Likert-type scales, careless responding, online surveys, survey responses, participant reactions

## Abstract

The effects of different response option orders on survey responses have been studied extensively. The typical research design involves examining the differences in response characteristics between conditions with the same item stems and response option orders that differ in valence—either incrementally arranged (e.g., strongly disagree to strongly agree) or decrementally arranged (e.g., strongly agree to strongly disagree). The present study added two additional experimental conditions—randomly incremental or decremental and completely randomized. All items were presented in an item-by-item format. We also extended previous studies by including an examination of response option order effects on: careless responding, correlations between focal predictors and criteria, and participant reactions, all the while controlling for false discovery rate and focusing on the size of effects. In a sample of 1,198 university students, we found little to no response option order effects on a recognized personality assessment vis-à-vis measurement equivalence, scale mean differences, item-level distributions, or participant reactions. However, the completely randomized response option order condition differed on several careless responding indices suggesting avenues for future research.

In the early 1930s, Rensis Likert presented the broad outlines of a psychological scaling method that was at the time thought to be more simple than other existing methods (Likert, 1932). The resulting adaption of Likert’s original concept is generally known as the Likert-type scale wherein respondents specify their level of agreement or disagreement (or frequency, importance, likelihood, etc.) on a symmetric scale for a series of statements (or items). After recoding reverse-scored items, participant responses to individual items are summed or averaged to arrive at a scale score. Over the past century, the Likert-type scale has been extremely popular across a wide range of fields such as education, psychology, business, polling, sociology, and public health.

Given its ubiquity, it is understandable that many studies have been conducted examining how Likert-type scales can be optimally utilized. A review of key Likert-type scale advances over the past quarter century was recently published (Jebb et al., 2021). They identified 40 studies that investigated the following areas related to Likert-type scale development: (a) conceptions of construct validity; (b) defining constructs; (c) creating scale items; (d) content validation; (e) conducting pilot studies; (f) measurement precision; (g) assessing factor structure; (h) creating short forms; and (i) empirical relations with other variables. Under (c) creating scale items, the subcategories were (1) readability tests; (2) modern readability measures; (3) respondent comprehension; (4) number of response options and labels; (5) item format; (6) item stability; and (7) presentation of items in blocks.

Jebb et al. (2021) only reviewed one study that examined item format (Zhang & Savalei, 2016), although there have been many other studies on item format that were not included in their review. Zhang and Savalei (2016) used an alternative scale format which replaced each response option in a Likert-type scale with a full sentence and found that such scales had better factor structure than traditional scales. Weigold et al. (2021) examined four item formats—horizontal radio button, text box, drop-down menu, and vertical radio button—and generally found both quantitative and qualitative equivalence across formats. Several studies have examined the effects of altering response option labels (e.g., only using verbal labels for the end points of the scale or removing verbal labels altogether; Gummer & Kunz, 2021; Spratto et al., 2021; Steinberg & Rogers, in press). The findings of this research generally support using verbal labels for each level of the response option continuum. In addition to the work of Weigold et al. (2021), several studies have examined the effect of horizontal versus vertical orientation of response options and the results of these studies have been inconclusive (see Hu, 2020 for a review of such studies). Additional item format issues have also been examined in other studies, such as the effects of polarity and verbalization of the middle category (Menold, 2021), and how to position and explain “don’t know” options on a survey (Zeglovits & Schwarzer, 2016).

To extend this existing body of research, the present study is focused on the effect of response option order on psychometric properties and participant reactions. Response option order refers to a type of item format in which the order of response options on Likert-type scales is presented differently (typically incrementally or decrementally in valence). We located 10 studies published over the past half century that have examined the effects of response option order on Likert-type survey responses (Chan, 1991; Christian et al., 2009; Garbarski et al., 2019; Höhne & Krebs, 2018; Krebs & Bachner, 2018; Krebs & Hoffmeyer-Zlotnik, 2010; Malhotra, 2008; Rammstedt & Krebs, 2007; Terentev & Maloshonok, 2019; Weng & Cheng, 2000). The results of these studies have been mixed as is illustrated by the summary in Table 1. All but one study found standardized differences (*d*) in means across response order conditions in the small range according to Cohen’s (1988) conventions (*d* < 0.20); standardized differences in means ranged from 0.04 to 0.18—except for Chan (1991) which found a standardized difference in means of 0.66. Differences in distributions as indexed by Cramer’s V ( $\phi_{c}$ ) across response option order were similarly in the small range (0.03–0.12) according to Cohen’s (1988) conventions ( $\phi_{c}<\;0.01$ ). Finally, again except for Chan (1991), factor structures were highly similar across response option order conditions. These research studies can be summarized as response option order typically evidencing rather small effects on mean differences, distributional differences, and differences in factor structures for Likert-type measures.^1^

**Table 1.** Summary of Studies Examining Effects of Response Option Order With Likert-Type Measures.

Study	Sample size	Means	Distributions	Factor structure
Chan (1991)	102	*d* = 0.66 (one scale-level comparison)	—	AGFI = 0.968 for decremental and AGFI = 0.799 for incremental
Weng and Cheng(2000)	858	$|\bar{d|}=0.10$ (two scale-level comparisons)	—	A change of ≤−.01 in CFI
Rammstedt and Krebs (2007)	315	$|\bar{d|}=0.04$ (five scale-level comparisons)	—	—
Malhotra (2008)	397	—	—	—
Christian et al. (2009)	810	$|\bar{d|}=0.08$ (10 item-level comparisons)	${\bar{\phi }}_{c}=0.10$ (10 item-level comparisons)	—
Krebs and Hoffmeyer-Zlotnik(2010)	436	$|\bar{d|}=0.18$ (eight scale-level comparisons)	only provided results for one of 13 item-level comparisons	Change of 0.00 in GFI, AGFI, and TLI and 0.08 in RMSEA
Höhne and Krebs(2018)	930	$|\bar{d|}=0.12$ (24 item-level comparisons)	—	Change of 0.00 in CFI and .006 in RMSEA
Krebs and Bachner(2018)	175 (Israeli) 250 (German)	$|\bar{d|}=0.15$ (Israeli sample) and $\bar{|d}|$ = 0.09 (German sample) for 12 item-level comparisons	${\bar{\phi }}_{c}=0.12$ (Israeli sample) and ${\bar{\phi }}_{c}=0.10$ (German sample) for three scale-level comparisons for each sample	Change of <0.01 in CFI and RMSEA for both samples
Garbarski et al.(2019)	2,945	—	$\phi_{c}=0.10$ (one item-level comparison)	—
Terentev andMaloshonok (2019)	22,910	—	${\bar{\phi }}_{c}=0.03$ (13 item-level comparisons)	—

*Notes.* All but Malhotra (2008) and Garbarski et al. (2019) utilized high school or university student samples. Malhotra (2008) did not present main effect statistics but instead examined whether primacy effects were modified by response time. They found that low-education respondents who filled out the questionnaire most quickly were most prone to primacy effects. *d* = Cohen’s standardized difference score effect size. Absolute values were used to compute average *d* scores so that positive and negative difference scores were appropriately combined. $\phi_{c}$ = Cramer’s *V* effect size. Average effect sizes are denoted with horizontal lines over the symbol. AGFI = adjusted goodness-of-fit index; CFI = comparative fit index; GFI = goodness-of-fit index; TLI = Tucker–Lewis Index; RMSEA = root mean square error of approximation; SA = strongly agree, SD = strongly disagree.

The present study is intended to extend the previous studies that have examined the effects of response option order on participant responses. In addition to replicating previous studies by examining the effects of response option order on mean differences and distributions, we have added several different dimensions to this research area. First, instead of simply examining incremental versus decremental response option orders, we have added two additional experimental conditions—randomly incremental versus decremental and totally randomized response option order. Second, because research has found careless responding to affect construct validity (see Kam, 2019), we investigate differences in careless responding across conditions and conduct all analyses using both minimal and thorough screening for careless responding. Third, because prediction is often a relevant concern when using attitudinal and personality variables, we examined whether response option order affected correlations with relevant outcomes. Fourth, we examined the effect of response option order on participant reactions. Particularly for the totally randomized condition, if participants find completing the questionnaire difficult or frustrating, this should be considered when deciding whether to implement its use from a practical perspective. Finally, we controlled for false discovery rate and focused heavily on effect sizes to ensure that our results were practically significant.

## Method

### Participants and Design

Participants consisted of 1,200 students from a large university located in southern Ontario, Canada with equal numbers of students assigned to each of the four experimental conditions. One student participated in two experimental conditions and was dropped from both conditions which reduced the sample size in two of the conditions to *n* = 299 (the overall sample size was reduced to 1,198). Remote online data collection using the Qualtrics survey software occurred from September 2020 to March 2021. The remote data collection was facilitated through the lead author’s institutional undergraduate business student research participation system. Students could earn extra credit for their courses by logging in to the system and selecting studies to participate in. If they chose to participate in our study, then they were directed to one of our survey’s Qualtrics pages (depending on their randomly assigned condition).

Most of the participants identified as female (52.3%), followed by male (47.5%), and other (0.2%) with an average age of 19.54 (*SD* = 1.22) years. Participants were mostly in their second year of studies (54.4%), followed by their third year of studies (45.6%), and the overwhelming majority were business majors (96.9%). Most of the participants identified as “not a visible minority” (52.3%) and reported that their native (i.e., first) language was English (72.3%). The remainder of the participants were highly fluent given that instruction at the university is exclusively in the English language and all students had successfully progressed to at least their second year.

Participants were randomly assigned to one of four conditions in which each item was presented on a separate page and the response options for the item were presented horizontally and arranged as follows: (a) incremental in valence from left to right (strongly disagree, disagree, neutral [neither agree nor disagree], agree, strongly agree); (b) decremental in valence from left to right (strongly agree, agree, neutral [neither agree nor disagree], disagree, strongly disagree); (c) randomly incremental or decremental in valence; and (d) totally randomized in valence for each item.^2^ Table 2 illustrates how each response option order was presented to participants.

**Table 2.** Illustrative Item Response Option Order(s) for Each Experimental Condition

Condition 1 (incremental response options)				
*Strongly disagree*	*Disagree*	*Neutral (neither agree nor disagree)*	*Agree*	*Strongly agree*
◯	◯	◯	◯	◯
Condition 2 (decremental response options)				
*Strongly agree*	*Agree*	*Neutral (neither agree nor disagree)*	*Disagree*	*Strongly disagree*
◯	◯	◯	◯	◯
Condition 3 (randomly incremental or decremental response options)				
*Strongly disagree*	*Disagree*	*Neutral (neither agree nor disagree)*	*Agree*	*Strongly agree*
◯	◯	◯	◯	◯
OR				
*Strongly agree*	*Agree*	*Neutral (neither agree nor disagree)*	*Disagree*	*Strongly disagree*
◯	◯	◯	◯	◯
Condition 4 (totally randomized response options)^a^				
*Neutral (neither agreenor disagree)*	*Agree*	*Disagree*	*Strongly agree*	*Strongly disagree*
◯	◯	◯	◯	◯
OR				
*Disagree*	*Strongly agree*	*Strongly disagree*	*Neutral(neither agreenor disagree)*	*Agree*
◯	◯	◯	◯	◯

Note: ^a^We provide two example response option orders in Condition 4 for illustrative purposes. There were 120 possible random permutations of response option order for this condition (5 × 4 × 3 × 2 × 1 = 120).

The order of presentation of the assessments in the survey was (a) HEXACO personality assessment; (b) participant reactions; (c) self-report of counterproductive academic behavior (CAB); and (d) demographic items (including self-report of grade point average [GPA]).

The manipulated (i.e., independent) variable in this design is response option order with all other variables acting as response (i.e., dependent) variables. In the case of the correlations between focal predictors (i.e., personality dimensions) and criterion variables (i.e., GPA and CAB), response option order is employed as a potential moderating variable.

### Measures

#### Personality Assessment

The 60-item version of the HEXACO Personality Inventory–Revised was utilized (https://hexaco.org/). The HEXACO assesses six major dimensions of personality: Honesty-Humility (H; sincere, honest, faithful, loyal, and modest/unassuming), Emotionality (E; emotional, oversensitive, sentimental, fearful, anxious, and vulnerable), Extraversion (X; outgoing, lively, sociable, talkative, cheerful, and active), Agreeableness (A; patient, tolerant, peaceful, mild, lenient, and gentle), Conscientiousness (C; organized, disciplined, diligent, careful, thorough, and precise), and Openness to Experience (O; intellectual, creative, unconventional, and innovative) (Ashton & Lee, 2009). An example item stem for Honesty-Humility is: “I would never accept a bribe, even if it were very large.” An example item stem for Conscientiousness is: “I often push myself very hard when trying to achieve a goal.”

Cross-condition internal consistency (α) coefficients in the present study were consistent with those found in Ashton and Lee (2009) for five of the six factors (with the reliability for H slightly lower): H (.69), E (.80), X (.80), A (.75), C (.78), and O (.74). Participants were given the following directions: “On the following pages you will find a series of statements about you. Please read each statement and decide how much you agree or disagree with that statement. Then choose your response to the statement using the scale provided. Please answer every statement, even if you are not completely sure of your response.”

#### Participant Reactions

Two questions were derived from Smither et al. (1993) for face validity and perceived predictive validity; one question was derived from Wiechmann and Ryan (2003) for liking. The items were: “The content of this questionnaire is clearly related to my study” (face validity); “With the results of this questionnaire my study performance can be predicted” (perceived predictive validity); and “I enjoyed completing this questionnaire” (liking). Participants responded on a 5-point Likert-type scale, ranging from “strongly disagree” to “strongly agree.” A supporting study was conducted by Holtrop et al. (2014) to estimate single-item reliability using the procedure by Wanous and Reichers (1996) of these three items, which found the following reliabilities: .55 for face validity, .60 for perceived predictive validity, and .64 for liking.^3^

We included two other participant reaction items: mental effort and fatigue (both using a 5-point Likert-type scale ranging from “strongly disagree” to “strongly agree”). Fatigue was measured with a single item: “I became fatigued and tired while working on this questionnaire” (Arvey et al., 1990). Mental effort was measured with a single item that was adapted from (Leppink et al., 2013): “Working on this questionnaire required me to exert a high level of mental effort.”

#### Outcome Variables Used to Judge Effects of Response Option Order on Criterion-Related Validity

##### Counterproductive Academic Behavior

We used the Holtrop et al. (2014) measure in which they extracted 25 items relevant to the school context from a 40-item inventory of counterproductive behaviors across school, home, and work contexts (Hakstian et al., 2002), using a 6-point Likert-type scale (“never even considered it”; “considered it, but didn’t do it”; “did it, perhaps once, but not sure”; “did it once”; “did it twice”; “did it three or more times”). An average total score across the 25 items representing overall CAB was used. An example item is: “During an exam, briefly glanced at another person’s paper.” The internal consistency (α) coefficient for this scale in the present study was acceptable (.86).

##### Grade Point Average

We asked students to self-report their GPA and we also asked students for their permission to access their official cumulative GPA from student records, which was a requirement from the research ethics board at the institution in which the data were sourced. The majority (58.1%) of participants consented to the use of their official cumulative GPA. We used self-report of GPA for the remaining participants. GPA scores ranged from 2.70 to 12 (D to A+; *M* = 9.55, *SD* = 1.33, which is an average of B+), with higher scores indicating better performance. The correlation between self-report and official GPA for those students who consented to providing us with their official GPA (*n* = 696) was *r* = .94 (*p* < .001; cf. the meta-analytic correlation estimate between self-report and official GPA for college students *r*_obs_ = .90, *SD*_obs_ = .05, *k* = 12, *N* = 12,089 from Kuncel et al., 2005). The standardized difference score between self-report and official GPA in our study was negligible (*d* = .02; cf. the meta-analytic standardized mean difference estimate between self-report and official GPA for college students *d* = 1.38, *SD*_obs_ = 0.46, *k* = 10, *N* = 6,507 from Kuncel et al., 2005). These results suggest that using self-report GPA (in lieu of official GPA) for part of our sample did not introduce a confound in the present study.

### Analyses

#### Careless Responding

The following screening criteria were used to remove participants: (a) answered that we should not use their data (i.e., self-reported data quality)^4^; (b) either did not answer or answered incorrectly for any of the three instructed response questions^5^; (c) a response time of <3 min for completion of the 63 items (60 HEXACO and three instructed response items)^6^; (d) longstring (i.e., maximum number of consecutive same responses) values at the elbow of a scree plot (Johnson, 2005); (e) an even-odd consistency index of <.30 (Jackson, 1977; Meade & Craig, 2012); (f) a Mahalanobis distance value above the 95th percentile (Desimone et al., 2015); and (g) an intra-individual response variability (IRV) value of ±2 *SD*s from the mean (Dunn et al., 2018; Marjanovic et al., 2015).

We computed one-way analyses of variance (ANOVAs) in SPSS between groups with Tukey’s HSD (honestly significant difference) post hoc tests (controlling for family-wise .05 alpha level) for each of the seven careless response indices we computed. We also computed standardized difference scores across conditions to gauge effect sizes for each dependent variable. The following careless responding indices were calculated: (a) even-odd consistency; (b) IRV; (c) longstring (item presentation order was randomized but we recovered the order presented to each participant using a Qualtrics export option and the R code as noted in the appendix)^7^; (d) Mahalanobis distance; (e) number of incorrect or missing instructed response answers; (f) time to completion (items were arranged one per survey page—we, therefore, used the total number of seconds the respondent spent on a given page, summed across items, and converted to minutes); and (g) number of missing responses.^8^ Analyses were aided by the R computing program package “careless” (Yentes & Wilhelm, 2018).

#### Screening for Careless Responding

We conducted all remaining analyses using (a) a *minimal* screening for careless responding (using #1 and #2 in screening criteria outlined above) and (b) a *thorough* screening for careless responding (using #1 through #7 in screening criteria outlined above) as careless responding has been found to affect psychometric properties (Kam, 2019). We predicted that differences for the remaining analyses across conditions would be more pronounced with minimal screening.^9^

#### Participant Reactions

We computed one-way ANOVAs in SPSS between groups with Tukey’s HSD post hoc tests (controlling for family-wise .05 alpha level) for each of the five participant reaction variables. We also computed standardized difference scores across conditions to gauge effect sizes for each variable.

#### Measurement Equivalence

We conducted multiple group confirmatory factor analyses (CFAs) in the R “lavaan” package comparing the four experimental conditions (Rosseel, 2012) using all six possible pairwise comparisons. Constraints were placed on parameters across groups to track successive levels of equivalence (configural invariance; metric invariance [factor loading and factor covariances]; scalar invariance) until fit was no longer adequate as judged by nonsignificant chi-square difference tests or negligible differences in the fit indices of comparative fit index (CFI) and root mean square error of approximation (RMSEA; models with CFI < .90 and RMSEA > .08 will be rejected). Hu and Bentler (1999) suggested cut-offs of .95 for CFI and .06 for RMSEA. These cut-off values are somewhat arbitrary (see Marsh et al., 2004) and the two indices can diverge (Lai & Green, 2016)—we, therefore, used the more liberal values of CFI > .90 and RMSEA < .08 to denote minimum levels of *acceptable* fit for any model (Kline, 2011). As the chi-square difference test is sensitive to sample size, we focused on changes in alternative fit indices such as CFI and RMSEA. Chen (2007) recommended that when the sample size was adequate and equal across groups, violation of measurement invariance should be indicated by “a change of ≤ −.01 in CFI, supplemented by a change of ≥.015 in RMSEA” (p. 501). Finally, we used diagonally weighted least squares (DWLS) estimation, which is appropriate for ordered categorical variables (Li, 2016).^10^

#### Criterion-Related Validity

We used SPSS programs found in Weaver and Wuensch (2013) to compute (a) the overall test (*Q*) of the equality of *k* independent correlations for each dependent variable (Fleiss, 1993; that is, two *Q* tests—one for each dependent variable); and (b) all possible comparisons of independent validity coefficients in the event a *Q* test is statistically significant at the .05 alpha level. We used the Benjamini and Hochberg (1995) false discovery rate algorithm to control for family-wise Type I error (at *p* < .05) of the test of two independent correlations with the family being considered the six comparisons for each dependent variable.^11^

## Results

### Careless Responding

Means and standard deviations for careless responding metrics across experimental conditions can be found in Table 3; the associated standardized mean differences with statistical significance tests (corrected for family-wise error) can be found in Table 4.

**Table 3.** Means and Standard Deviations for Careless Responding Metrics Across Experimental Conditions

Careless responding metric	Condition 1		Condition 2		Condition 3		Condition 4	
	*M*	*SD*	*M*	*SD*	*M*	*SD*	*M*	*SD*
Even-odd consistency	0.66	0.44	0.67	0.43	0.66	0.45	0.65	0.44
Intra-individual response variability	1.15	0.20	1.18	0.18	1.19	0.20	1.39	0.08
Longstring index	4.71	5.47	4.06	1.80	4.42	2.60	3.35	0.91
Mahalanobis distance	55.49	19.37	58.66	21.18	58.73	19.85	66.93	25.91
Number of incorrect directed responses	0.10	0.38	0.14	0.48	0.06	0.30	0.11	0.46
Time to completion (min)	11.74	24.35	11.88	26.19	12.57	36.03	11.52	13.96

*Notes. N* = 1,198 for careless responding metrics (approximate *n* = 300 per condition). Condition 1 = incremental response options (SD to SA). Condition 2 = decremental response options (SA to SD). Condition 3 = randomly incremental or decremental response options. Condition 4 = totally randomized response options. Three directed response items were utilized (i.e., score can vary from 0 to 3). SA = strongly agree, SD = strongly disagree.

**Table 4.** Standardized Mean Differences for Careless Responding Metrics Across Experimental Conditions.

Careless responding metric	*d*_1–2_	*d*_1–3_	*d*_1-4_	*d*_2–3_	*d*_2–4_	*d*_3–4_
Even-odd consistency	–0.02	0.00	0.02	0.02	0.05	0.02
Intra-individual response variability^a^	−0.16	−0.20	−1.58*	−0.05	−1.51*	−1.31*
Longstring index^a^	0.16	0.07	0.35*	−0.16	0.50*	0.55*
Mahalanobis distance^a^	−0.16	−0.17	−0.50*	0.00	−0.35*	−0.36*
Number of incorrect directed responses^a^	−0.09	0.12	−0.02	0.20	0.06	−0.13
Time to completion (min)	−0.01	−0.03	0.01	−0.02	0.02	0.04

*Notes. N* = 1,198 (approximate *n* =300 per condition). Condition 1 = incremental response options (SD to SA). Condition 2 = decremental response options (SA to SD). Condition 3 = randomly incremental or decremental response options. Condition 4 = totally randomized response options.

* *p* < .05 using Tukey’s honestly significant difference post hoc test (or Tamhane’s T2 post hoc test when variances are unequal across conditions—noted by superscript [^a^]). SA = strongly agree, SD = strongly disagree.

The preregistered expectations were as follows: (a) Conditions 1 and 2 are not expected to differ on the careless responding metrics as they are very similar in format; (b) Conditions 1 and 2 are expected to have higher longstrings than Conditions 3 and 4 because the consistent format will be more conducive to repeated consecutive responses; (c) Condition 3 will have higher longstrings than Condition 4 because the randomized nature of the fourth condition would make it difficult to easily repeat consecutive responses; (d) Conditions 3 and 4 are expected to have longer time to complete because the change in response option order from item to item will slow reading speed; and (e) Condition 4 is expected to have longer time to complete than Condition 3 because totally randomized response options are likely less easy to process than response option orders that are simply flipped in a linear fashion from item to item. Expectation (a) was supported. Expectation (b) was partially supported; however, Condition 3 did not have a higher longstring than Conditions 1 or 2. Expectation (c) was supported in that Condition 3 had a higher longstring index than Condition 4. Expectations (d) and (e) were not supported in that there were no differences in time to completion across conditions. The differences in longstring indices that were statistically significant were medium in size (average *d*≈ 0.5).^12^

Several non-preregistered significant differences were found during exploratory follow-up analyses. Participants in Conditions 1, 2, and 3 evidenced lower IRVs than in Condition 4, and the effect sizes were very large (average *d*≈ 1.5). The average Mahalanobis distances were significantly higher in the fourth condition compared with the other three conditions, and these differences were in the small to medium range (*d* values ranged from .35 to .50).

### Screening for Careless Responding

Interestingly, even though the conditions differed significantly on several of the careless responding metrics, the numbers of participants screened out through minimal and thorough screens for careless responding did not significantly differ across conditions using the Z-test for differences in proportions. Approximately 10% of participants within each condition were screened out using the minimal screening criteria (which resulted in the following numbers of remaining participants in each condition: *n*_1_ = 272, *n*_2_ = 266, *n*_3_ =268, *n*_4_ = 270). Approximately 30% of participants within each condition were screened out using the thorough screening criteria (which resulted in the following numbers of remaining participants in each condition: *n*_1_ = 209, *n*_2_ = 207, *n*_3_ = 195, *n*_4_ = 199). We conjecture that the lack of differences across conditions in number of participants screened out due to careless responding was the result of the higher average means for the longstring index canceling out the higher average IRV and Mahalanobis distance values in Conditions 1, 2, and 3 compared with Condition 4.

### Participant Reactions

The means and standard deviations for participant reactions across experimental conditions can be found in Table 5; the associated standardized mean differences with statistical significance tests (corrected for family-wise error) can be found in Table 6. The pre-registered expectations were as follows: (a) Conditions 1 and 2 are not expected to differ on any of the reaction measures; (b) Conditions 3 and 4 are expected to have more negative reactions on “liking” than Conditions 1 and 2; and (c) Condition 4 is expected to have more negative reactions on “liking” than Condition 3. Expectation (a) was supported. Expectations (b) and (c) were not supported; in fact, the only statistically significant effect for participant reactions was that Condition 4 was judged to have higher face validity than Condition 2 in the minimal careless responding condition, but this effect was not found with thorough screening.

**Table 5.** Means and Standard Deviations for Participant Reactions Across Experimental Conditions.

Variable	Condition 1		Condition 2		Condition 3		Condition 4	
	*M*	*SD*	*M*	*SD*	*M*	*SD*	*M*	*SD*
Face validity^a^	2.71	0.95	2.64	0.94	2.78	0.98	2.86	1.01
Face validity^b^	2.74	0.95	2.67	0.91	2.73	0.93	2.87	0.96
Perceived predictive validity^a^	2.87	1.02	3.01	1.03	2.97	0.95	2.98	1.01
Perceived predictive validity^b^	2.85	1.00	3.00	1.01	3.02	0.92	3.06	0.94
Liking^a^	3.66	0.68	3.68	0.77	3.78	0.79	3.71	0.80
Liking^b^	3.66	0.65	3.70	0.72	3.75	0.74	3.72	0.74
Mental effort^a^	2.28	0.93	2.28	0.86	2.34	1.27	2.30	0.90
Mental effort^b^	2.23	0.93	2.24	0.85	2.33	1.14	2.28	0.84
Fatigue^a^	2.07	0.88	2.02	0.90	2.08	0.95	1.98	0.85
Fatigue^b^	2.00	0.80	2.00	0.91	2.07	0.88	1.93	0.74

*Notes.* ^a^*N* = 1,076 for minimal careless responding screening (approximate *n* = 270 per condition). ^b^*N* = 810 for thorough careless responding screening (approximate *n* = 200 per condition). Condition 1 = incremental response options (SD to SA). Condition 2 = decremental response options (SA to SD). Condition 3 = randomly incremental or decremental response options. Condition 4 = totally randomized response options. SA = strongly agree, SD = strongly disagree.

**Table 6.** Standardized Mean Differences for Participant Reactions Across Experimental Conditions.

Variable	*d*_1–2_	*d*_1–3_	*d*_1–4_	*d*_2–3_	*d*_2–4_	*d*_3–4_
Face validity^a^	0.07	−0.07	−0.15	−0.15	−0.23*	−0.08
Face validity^b^	0.08	0.01	−0.14	−0.07	−0.21	−0.15
Perceived predictive validity^a^	−0.14	−0.10	−0.11	0.04	0.03	−0.01
Perceived predictive validity^b^	−0.15	−0.18	−0.22	−0.02	−0.06	−0.04
Liking^a^	−0.03	−0.16	−0.07	−0.13	−0.04	0.09
Liking^b^	−0.06	−0.13	−0.09	−0.07	−0.03	0.04
Mental effort^a^	0.00	−0.05	−0.02	−0.06	−0.02	0.04
Mental effort^b^	−0.01	−0.10	−0.06	−0.09	−0.05	0.05
Fatigue^a^	0.06	−0.01	0.10	−0.06	0.05	0.11
Fatigue^b^	0.00	−0.08	0.09	−0.08	0.08	0.17

*Notes.* ^a^*N* = 1,076 for minimal careless responding screening (approximate *n* = 270 per condition). ^b^*N* = 810 for thorough careless responding screening (approximate *n* = 200 per condition). Condition 1 = incremental response options (SD to SA). Condition 2 = decremental response options (SA to SD). Condition 3 = randomly incremental or decremental response options. Condition 4 = totally randomized response options. *p < .05 using Tukey’s honestly significant difference post hoc test. Results in this table were substantively similar when the nonparametric independent-samples Kruskal–Wallis Tests with Bonferroni corrections were computed. SA = strongly agree, SD = strongly disagree.

### Measurement Equivalence

The full CFA results for both the minimally and thoroughly screened participants can be found in the supplementary material. The fit statistics for the baseline (i.e., configural invariance) model for minimally screened participants were all acceptable (*M*_CFI_ = .905, *SD*_CFI_ = .006; *M*_RMSEA_ = .054, *SD*_RMSEA_ = .002); as were those for thoroughly screened participants (*M*_CFI_ = .927, *SD*_CFI_ = .007; *M*_RMSEA_ = .052, *SD*_RMSEA_ = .001). Furthermore, the CFA results suggested a high level of metric and scalar^13^ invariance across conditions for both minimally and thoroughly screened participants in that in no comparisons did a change of ≤−.01 in CFI, supplemented by a change of ≥.015 in RMSEA occur.

The results of the non-preregistered tests of significance for the internal consistency (alpha) coefficients across experimental conditions can be found in Table 7. None of the internal consistency coefficients were significantly different from one another across conditions at the .05 alpha level (even before correcting for family-wise error).

**Table 7.** Internal Consistency (Alpha) Coefficients Across Experimental Conditions for the HEXACO Personality Scales.

Scale	Condition 1	Condition 2	Condition 3	Condition 4
Minimal careless responding screening				
Honesty-Humility (H)	.73	.68	.71	.69
Emotionality (E)	.79	.81	.82	.80
Extraversion (X)	.83	.80	.82	.81
Agreeableness (A)	.72	.75	.77	.78
Conscientiousness (C)	.79	.79	.77	.76
Openness to experience (O)	.76	.78	.74	.75
Thorough careless responding screening				
Honesty-Humility (H)	.76	.72	.73	.72
Emotionality (E)	.81	.82	.83	.84
Extraversion (X)	.86	.81	.84	.85
Agreeableness (A)	.74	.78	.80	.79
Conscientiousness (C)	.81	.78	.79	.79
Openness to experience (O)	.75	.79	.75	.76

*Notes.* These analyses were not pre-registered. *N* = 1,076 for minimal careless responding screening (approximate *n* = 270 per condition). *N* = 810 for thorough careless responding screening (approximate *n* = 200 per condition). Condition 1 = incremental response options (SD to SA). Condition 2 = decremental response options (SA to SD). Condition 3 = randomly incremental or decremental response options. Condition 4 = totally randomized response options. None of the internal consistency coefficients were significantly different from one another across conditions at the .05 alpha level. SA = strongly agree, SD = strongly disagree.

### Criterion-Related Validity

We did not make any pre-registered expectations for differences in criterion-related validity across experimental conditions. The correlations between focal (H and C HEXACO scales) and criterion (GPA and CAB) variables can be found in Table 8 for both minimal and thorough screening for careless responding. The only statistically significant difference was between Conditions 2 and 3 (*r* = −.40 vs. *r* = −.15, respectively) with Honesty-Humility as the predictor and CAB as the criterion when participants were minimally screened for careless responding. This effect was nullified when participants were screened thoroughly for careless responding.

**Table 8.** Correlations Between Focal Predictor and Criterion Variables.

*Q*	*df*	*p*	Condition 1	Condition 2	Condition 3	Condition 4
Conscientiousness → GPA (minimal careless responding screening) (approximately *n* = 270)						
0.36	3	.948	.20**	.21***	.23***	.18**
Conscientiousness → GPA (thorough careless responding screening) (approximately *n* = 200)						
4.01	3	.260	.26***	.25***	.32***	.13
Honesty-Humility → CAB (minimal careless responding screening) (approximately *n* = 270)						
9.79	3	.020	−.29***	−.40***	−.15*	−.28***
Honesty-Humility → CAB (thorough careless responding screening) (approximately *n* = 200)						
5.90	3	.117	−.33***	−.43***	−.21**	−.33***

*Notes*. Condition 1 = incremental response options (SD to SA). Condition 2 = decremental response options (SA to *SD*). Condition 3 = randomly incremental or decremental response options. Condition 4 = totally randomized response options. Underlined values are significantly different at the .05 alpha level correcting for false discovery rate. GPA = cumulative grade point average; CAB = counterproductive academic behavior. SA = strongly agree, SD = strongly disagree.

* *p* < .05. **p < .01. ***p < .001.

## Discussion

The results of the present study generally show little effect for response option order in a sample of university students on a recognized measure of personality. Little to no differences were found across conditions in participant reactions, measurement equivalence, scale mean differences, item distributions, or correlations between focal predictors and criterion variables. The results related to measurement equivalence, scale mean distributions, and item distributions are largely consistent with previous research that has found few practically significant effects. The study by Chan (1991) was the only one of the 10 we reviewed that showed a practically significant effect of response option order on mean differences and measurement equivalence. However, this study should be viewed with caution due to its relatively small sample size (*N* = 102), its utilization of a survey translated from English to Mandarin (a pictogram-based alphabet likely limits generalizability to a letter-based alphabet), and the fact that a within-subjects design was used with the decremental response option order always presented first (i.e., no counter-balancing of response option order that may have led to carryover effects).

Three of the six careless responding indices *did* show both practically and statistically significant differences across conditions. Specifically, IRV means and Mahalanobis distance means were lower and longstring index means were higher for the nonrandomized response option conditions versus the totally randomized response option condition. One would assume that higher IRV and Mahalanobis values would suggest that error variance is increasing, which, in turn, would lead to degradation in measurement equivalence, lower correlations between focal predictors and criterion, and perhaps differences in item-level distributions. However, our results do not support such a causal sequence. This finding was particularly interesting as previous research has suggested that careless responding degrades construct validity (cf. Kam, 2019).

Straightlining is when survey respondents give identical or nearly identical answers to items on a multi-item survey on the same response scale (Kim et al., 2019). In our cumulative consulting experience, the issue of straightlining has been of great practical importance to our clients who want to ensure that data quality is not degraded by the phenomenon; academicians who conduct survey-based research would also be likely to echo this sentiment. In our study, straightlining was reduced in the completely randomized response option condition with small to medium effect sizes. A future study should experimentally examine the differences in straightlining between a grid format (where items are presented in rows on the same page and sharing a common set of response options) using both incremental and decremental response option orders and an item-by-item format using a completely randomized response option order. In addition to possible differences in psychometric properties across these conditions, it would be interesting to examine whether respondents in a real-life, applied context (e.g., managers rating employee performance or individuals completing actual structured quantitative reference checks) would have more negative reactions to a completely randomized response order than was found in this study.

In our review of studies in this area, it is apparent that researchers in this area have focused heavily on using null hypothesis significance testing (NHST) even though experts in the social sciences have warned against sole reliance on this approach (cf. Cohen, 1994). None of the 10 studies that we reviewed reported effect sizes (we computed them post hoc). Moreover, almost all of the studies made multiple statistical comparisons which can lead to inflated Type I error. Again, none of the 10 studies attempted to control for this inflated Type I error by controlling for false discovery rate (cf. Benjamini & Hochberg, 1995). The present study is unique in that we focused on effect sizes and controlled for false discovery rate. Future studies in this area should follow our lead so that we can be more confident in the existence and size of effects related to response option order.

In conclusion, the results of the present study do not suggest that response option order should be of particular concern to those building assessments. However, more research is likely necessary to ensure that our findings generalize to other contexts, different types of assessments, the use of a horizontal versus vertical orientation (see Garbarski et al., 2019), and using different comparisons (e.g., grid format to item-by-item format). Finally, the results of differences in careless responding indices for the completely randomized response option order condition in comparison with the other conditions suggest several avenues for future research.

## Supplemental Material

sj-xlsx-1-epm-10.1177_00131644211069406 – Supplemental material for Effects of Response Option Order on Likert-Type Psychometric Properties and Reactions Click here for additional data file. Supplemental material, sj-xlsx-1-epm-10.1177_00131644211069406 for Effects of Response Option Order on Likert-Type Psychometric Properties and Reactions by Chet Robie, Adam W. Meade, Stephen D. Risavy and Sabah Rasheed in Educational and Psychological Measurement

sj-xlsx-2-epm-10.1177_00131644211069406 – Supplemental material for Effects of Response Option Order on Likert-Type Psychometric Properties and Reactions Click here for additional data file. Supplemental material, sj-xlsx-2-epm-10.1177_00131644211069406 for Effects of Response Option Order on Likert-Type Psychometric Properties and Reactions by Chet Robie, Adam W. Meade, Stephen D. Risavy and Sabah Rasheed in Educational and Psychological Measurement

## Appendix

**R code to reorder randomly ordered questions from Qualtrics survey software to the order presented to each participant**

reorderHelper <- function(orderArray,responseArray){

outArray = rep(NA, length(responseArray)) ## make a an array of NAs

for(i in 1:length(orderArray)){ # loop the order array

 location <- orderArray[i] # get the admin location

 outArray[location] = responseArray[i] # get the next response in the array and write it to the correct spot in the output array

 }

 return(outArray)

}

reorderMain <- function(orderDF,responseDF){

 outDF <- responseDF # dimension and name the output df

 outDF[,] <- NA #replace with NA for safety

 for(j in 1:nrow(orderDF)){ # loop all rows of dataframe

 orderArr <- as.numeric(orderDF[j,]) # take row and convert to array

 respArr <- as.numeric(responseDF[j,]) # take row and convert to array

 outDF[j,] <- reorderHelper(orderArr, respArr) # reorderHelper does the reodering and result is written here

 }

 return(outDF)

}

#### create a dataframe of the responses provided by Qualtrics named rDF#### create a dataframe of the item order provided by Qualtrics named oDF

reordered.df <- reorderMain(oDF,rDF)

**R code to reorder randomly ordered questions and response options from Qualtrics survey software to the order presented to each participant**

# functions for reordering response options

findLocation <- function(arr){

 location <- NA # default to NA

 resp <- arr[1] # first element is the response itself

 theOrderArr <- arr[2:length(arr)] # the rest of the array is the order list

 location <- match(resp,theOrderArr) # finds the location in the array of response

}

getResponseOptionLocation <- function(dfRow){ # takes a single row of a df

 nColPerItem <- 6 ### with 5 response options, Qualtrics provides 6 columns of information per item. The response as well as the order of presentation of the 5 response options

 nItems <- ncol(dfRow) / nColPerItem # modify

 outputArray <- array (NA, dim=c(nItems)) # initialize output

 outCounter <- 1 # initial output counter

 for (i in seq(from=1, to=ncol(dfRow), by= nColPerItem)){

 # start column is i, end col

 endCol <- i + 5 # assumes 5 response options

 thisItemArray <- as.numeric(dfRow[,i:endCol]) # get one item and convert to numeric array

 outputArray[outCounter] <- findLocation(thisItemArray) # call the function to do the work

 outCounter <- outCounter + 1

 }

 return(outputArray)

}

### change your file names as needed below

#oDF is the name of the original dataframe that contains only the item responses

#d.1 is the dataframe with Qualtrics information about item order presentation

rDF <- oDF # dimension and name the output df

rDF[,] <- NA #replace with NA for safety

for (j in seq(from=1, to=nrow (d.1))){

 rDF[j,] <- getResponseOptionLocation(d.1[j,])

}

## Notes

1. It should be noted that effects of category order with non-Likert-type measurement have been similarly mixed. Christian et al. (2009) and Holbrook (2008) provide brief reviews of such studies.

2. We only used verbal anchors as previous research (cf. Rammstedt & Krebs, 2007) has found some evidence to suggest that adding numerical anchors could act as a confound.

3. The Wanous and Reichers (1996) method for estimating the reliability of a single-item measure requires correlating a single-item measure with a multi-item measure of the same construct and then using Nunnally and Bernstein’s (1994, p. 257) correction for attenuation equation to solve for its missing value in the denominator (i.e., the reliability for the single-item measure).

4. Relatively few individuals reported that we should not use their data (<5% of the total sample): Condition 1 (*n* = 10); Condition 2 (*n* = 9); Condition 3 (*n* = 20); Condition 4 (*n* = 14)—these differences across conditions were not statistically significant χ^2^(3) = 5.96, *p* =.11, $\phi_{c}=0.07$ .

5. The three directed response item stems were: “Please respond with ‘strongly disagree’ for this item”; “Please respond with ‘neutral (neither agree nor disagree)’ for this item”; and “Please respond with ‘agree’ for this item.”

6. One of the authors (with high reading speed and who has taken many similar personality measures over the course of their career) attempted the 63 items (HEXACO and instructed response items) for all four conditions in as fast a pace as possible while still maintaining accuracy. The response times to complete each version were: Condition 1 (3.52 min); Condition 2 (3.52 min); Condition 3 (3.32 min); and Condition 4 (3.80 min).

7. Qualtrics exports the data in standard order (not the random order presented to each participant) but does provide the order that the items were presented to each participant in a separate string. Computer code needed to be written to use the order from this separate string to reorder the data so that the longstring could be computed (see the appendix for the R code).

8. We did not present the results for the number of missing responses as there was only one missing response in the second condition and one missing response in the third condition.

9. We did not use the counterproductive academic behavior items to calculate careless responding metrics for #5, #6, #7, or #8 as the low base rates of such behavior may necessarily create unusual psychometric properties that may falsely flag a participant exerting sufficient effort as careless.

10. Although not preregistered, we also computed tests of statistical significance for the internal consistency (α) coefficients across groups for all the HEXACO personality scales using the cocron R package (Diedenhofen & Musch, 2016).

11. We only examined H and C for measurement equivalence analyses because power analyses suggested sample sizes of 100 to 300 for two latent factors with 10 indicators per factor (Nicolaou & Masoner, 2013; Wolf et al., 2013). We similarly restricted criterion-related validity differences across conditions to H and C as a fair comparison of whether any lack of measurement equivalence is matched to differences in criterion-related validity.

12. According to the conventions outlined by Cohen (1988) of .2, .5, and .8 being indicative of small, medium, and large effect sizes, respectively.

13. We computed one-way analyses of variance (ANOVAs) on all six of the HEXACO scales with the scales as the dependent variables and experimental condition as the independent variable on both minimally and thoroughly screened data. None of the manifest means were significantly different from one another for any of the scales using Tukey HSD (honestly significant difference) post hoc tests. The average absolute value difference score ( $\left|\bar{d|}\right)$ comparing the HEXACO scales for Condition 1 versus the other three conditions for minimally and thoroughly screened data (a total of 36 comparisons) was 0.06 (*SD* = 0.05) which is considered a small effect (*d* < 0.20, Cohen, 1988). We also computed Cramer’s *V* ( $\phi_{c}$ ) coefficients to examine the degree to which response patterns differed at the item level across conditions. For both minimally and thoroughly screened data the mean $\phi_{c}$ effect size across the 60 items on the HEXACO personality assessment was 0.07 (*SD* = 0.01), which is also considered a small effect ( $\phi_{c}<\;0.10$ ; Cohen, 1988). Moreover, only seven and four of the 60 $\phi_{c}$ coefficients were statistically significant at the .05 alpha level for minimal screening and thorough screening, respectively. These analyses were not preregistered.
